# Triptolide suppresses the growth and metastasis of non-small cell lung cancer by inhibiting β-catenin-mediated epithelial–mesenchymal transition

**DOI:** 10.1038/s41401-021-00657-w

**Published:** 2021-04-23

**Authors:** Qiu-di Deng, Xue-ping Lei, Yi-hang Zhong, Min-shan Chen, Yuan-yu Ke, Zhan Li, Jing Chen, Li-juan Huang, Yu Zhang, Lu Liang, Zhong-xiao Lin, Qing Liu, Song-pei Li, Xi-yong Yu

**Affiliations:** 1 GMU-GIBH Joint School of Life Sciences, Guangzhou Medical University, Guangzhou, 511436 China; 2 Key Laboratory of Molecular Target & Clinical Pharmacology and the State Key Laboratory of Respiratory Disease, School of Pharmaceutical Sciences & The Fifth Affiliated Hospital, Guangzhou Medical University, Guangzhou, 511436 China; 3 College of Pharmacy, Xiangnan University, Chenzhou, 423000 China

**Keywords:** non-small cell lung cancer, metastasis, migration, invasion, triptolide, epithelial–mesenchymal transition, β-catenin

## Abstract

Non-small cell lung cancer (NSCLC) is characterized by a high incidence of metastasis and poor survival. As epithelial–mesenchymal transition (EMT) is well recognized as a major factor initiating tumor metastasis, developing EMT inhibitor could be a feasible treatment for metastatic NSCLC. Recent studies show that triptolide isolated from *Tripterygium wilfordii* Hook F attenuated the migration and invasion of breast cancer, colon carcinoma, and ovarian cancer cells, and EMT played important roles in this process. In the present study we investigated the effect of triptolide on the migration and invasion of NSCLC cell lines. We showed that triptolide (0.5, 1.0, 2.0 nM) concentration-dependently inhibited the migration and invasion of NCI-H1299 cells. Triptolide treatment concentration-dependently suppressed EMT in NCI-H1299 cells, evidenced by significantly elevated E-cadherin expression and reduced expression of ZEB1, vimentin, and slug. Furthermore, triptolide treatment suppressed β-catenin expression in NCI-H1299 and NCI-H460 cells, overexpression of β-catenin antagonized triptolide-caused inhibition on EMT, whereas knockout of β-catenin enhanced the inhibitory effect of triptolide on EMT. Administration of triptolide (0.75, 1.5 mg/kg per day, ip, every 2 days) for 18 days in NCI-H1299 xenograft mice dose-dependently suppressed the tumor growth, restrained EMT, and decreased lung metastasis, as evidence by significantly decreased expression of mesenchymal markers, increased expression of epithelial markers as well as reduced number of pulmonary lung metastatic foci. These results demonstrate that triptolide suppresses NSCLC metastasis by targeting EMT via reducing β-catenin expression. Our study implies that triptolide may be developed as a potential agent for the therapy of NSCLC metastasis.

## Introduction

Lung cancer ranks first among all malignancies in cancer-related deaths worldwide. Non-small cell lung cancer (NSCLC) accounts for ~85% of lung carcinomas and is classified into squamous-cell carcinoma, adenocarcinoma (AC), and large-cell carcinoma [1–3]. Although many specific and effective therapeutic strategies (including surgery, chemoradiotherapy, immunotherapy, and molecular targeted therapy) have been investigated for NSCLC, NSCLC patients still exhibit poor prognosis. Tumor metastasis has been recognized as one of the major factors leading to the therapeutic failure of NSCLC. Approximately 80%–90% of NSCLC patient deaths are attributed to metastasis [4, 5]. Thus, identification of molecular targets and development of specific and effective agents for metastatic NSCLC therapy are urgently needed.

Aberrant epithelial-to-mesenchymal transition (EMT) is a major factor initiating tumor metastasis. EMT is a dynamic process by which epithelial cells lose epithelial properties (such as loss of cell–cell contacts and polarity and a decrease in the epithelial marker E-cadherin) and present mesenchymal characteristics (such as elevation of the mesenchymal markers N-cadherin, vimentin, and fibronectin). EMT facilitates tumor metastasis by allowing cancer cells to disseminate from the primary tumor, intravasate, circulate, and eventually extravasate to other organs [6–8]. Increasing evidence has shown that EMT is critical for the invasiveness and metastasis of NSCLC. E-cadherin expression decreased in NSCLC tissues, whereas the expression of mesenchymal markers, including N-cadherin and vimentin, was upregulated in NSCLC tissues. In addition, N-cadherin and vimentin have been proposed as potential predictors for patient survival. Several EMT-inducing transcription factors, including ZEB1 and Twist, have been suggested to be involved in NSCLC metastasis [9–12]. Suppression or reversal of EMT significantly reduced the migration and invasiveness of NSCLC cells, and EMT has therefore become a potential therapeutic target for NSCLC metastasis [8, 13, 14]. Identifying agents that can reverse or suppress EMT is likely to be an effective way to develop pharmaceuticals for the suppression of NSCLC metastasis.

**Fig. 1:**
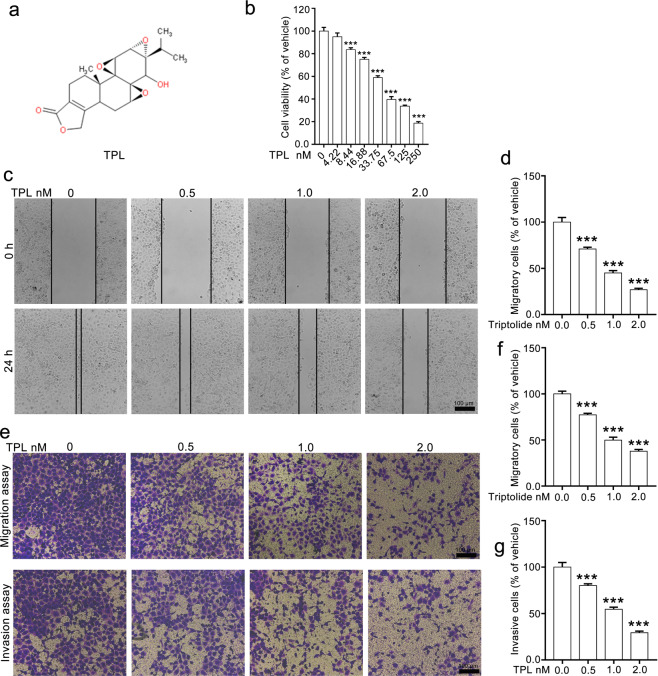
Triptolide restrains the migration and invasion of NCI-H1299 cells. **a** The chemical structural formula of triptolide. **b** Triptolide suppressed the proliferation of NCI-H1299 cells in a dose-dependent manner. **c**, **d** The effect of triptolide on the migration of NCI-H1299 cells determined by wound-healing assays. **e**, **f** The effect of triptolide on the migration and invasion of NCI-H1299 cells determined by Transwell migration and invasion assays. Representative images (×100 magnification) are shown in **e**. Quantitative data were calculated using ImageJ, analyzed by using GraphPad Prism 5.0 and are shown in **f** and **g**. TPL triptolide. The data are presented as the mean ± SEM, *n* = 3. ***P < 0.001 vs. the vehicle group.

Pharmacologically active compounds derived from Chinese herbal medicine are critical sources of anticancer drugs, and many of them have been applied in clinical or preclinical trials [15, 16]. Triptolide (Fig. 1a) is isolated from *Tripterygium wilfordii* Hook F, which has been widely used for the treatment of autoimmune disorders and inflammation, such as lupus and rheumatoid arthritis, in traditional Chinese medicine. Commercial preparations of triptolide are used clinically for the treatment of rheumatoid arthritis, systemic lupus erythematosus, nephritis, and psoriasis [17, 18]. Triptolide also shows potent antitumor effects in a variety of solid tumors (breast cancer, liver cancer, lung cancer, and pancreatic cancer) by inducing cell cycle arrest and apoptosis. Heat shock protein (HSP), the NF-κB pathway, and the caspase signaling pathway have been reported to mediate the antitumor effect of triptolide [19–22]. Recently, several studies have shown that triptolide attenuated the migration and invasion of breast cancer cells, colon carcinoma cells, and ovarian cancer cells, and EMT played important roles in this process [23–25]. Triptolide has also been reported to suppress tumor growth by inhibiting the proliferation and self-renewal of pulmospheres [26]. However, whether it suppresses NSCLC metastasis and the underlying mechanism have not been fully elucidated.

In the present study, we investigated the effect of triptolide on the migration and invasion of NCI-H1299 cells and found that triptolide strongly suppressed the migration, invasion, EMT, and metastasis of NSCLC cells in vitro and in vivo by downregulating β-catenin expression. Our study provides scientific evidence for developing triptolide as a potential agent for metastatic NSCLC therapy.

## Materials and methods

### Materials

Triptolide with a purity of 98% was supplied by SelleckChem (Houston, TX, USA) and then dissolved in DMSO to produce a 20 mM stock solution. Fetal bovine serum (FBS), penicillin-streptomycin, Lipofectamine 3000 reagent, Lipofectamine LTX&PLUS reagent and NE-PER nuclear and cytoplasmic extraction reagents were purchased from Thermo Fisher Scientific (Waltham, MA, USA). Twenty-four-well Transwell plates (6.5 mm) with 8.0 μm polycarbonate membrane inserts and Matrigel were obtained from Corning (New York, NY, USA). A Cell Counting Kit (CCK-8) was purchased from Yeasen Biotech (Shanghai, China). Antibodies against β-catenin, N-cadherin, vimentin, snail, slug, E-cadherin, ZO-1, β-actin and Lamin B and Alexa Fluor 594 donkey anti-rabbit IgG and HRP-conjugated anti-rabbit IgG antibodies were purchased from Cell Signaling Technology (Danvers, MA, USA). The pCMV3-CTNNB1-GFP and pCMV3-GFP vectors were purchased from Sino Biological, Inc. (Beijing, China). TRIzol reagent and 4′,6-diaminido-2-phenylindole dihydrochloride (DAPI) were obtained from Invitrogen (Carlsbad, CA, USA). The Transcriptor First Strand cDNA Synthesis kit and One-Step SYBR PrimeScript Plus quantitative real-time reverse transcriptase-polymerase chain reaction (RT-qPCR) kit were purchased from TaKaRa (Kyoto, Japan). Recombinant human Wnt3a was obtained from R&D Systems, Inc. (Minneapolis, MN, USA). LiCl was obtained from Millipore Sigma (Darmstadt, Germany). MG132 and other reagents were purchased from Sigma-Aldrich (St. Louis, MO, USA).

### Cell culture

The NSCLC cell lines NCI-H1299 and NCI-H460 were obtained from the American Type Culture Collection (Manassas, VA, USA). The cells were maintained in DMEM supplemented with 10% FBS (v/v) and 1% penicillin-streptomycin (v/v) and then incubated at 37 °C in a humidified incubator containing 5% CO_2_. The cells tested negative for mycoplasma using the STR Multi-Amplification Kit (Microreader 21 ID System).

### Animals

Healthy Kunming mice of both sexes (weighing ~25 g) and four- to five-week-old male *nu/nu* BALB/c mice were obtained from Hua Fukang Experimental Animal Center (Beijing, China). All the experimental procedures were previously approved and supervised by the Animal Experiment Ethics Committee of Guangzhou Medical University (Guangzhou, China) according to ARRIVE guidelines.

### Cell proliferation assay

Cell viability was detected by CCK-8 assays. Briefly, cells (5 × 10^3^ cells/well) were seeded in 96-well plates and cultured for 24 h, followed by exposure to various concentrations of triptolide for 48 h. Ten microliters of CCK-8 reagent was added to each well and incubated for another 2 h, and optical density values at 450 nm were detected by using a microplate reader. For each experiment, three independent runs were performed to calculate the standard error of the mean (SEM).

### Scratch wound-healing assay

Cells (3 × 10^5^ cells/well) were seeded in six-well plates and cultured overnight to form a confluent monolayer. The cells were starved with serum-free medium for 12 h and scratched with a sterile 10-μL micropipette tip to create a straight line as a wound. After removal of the dead cells, the attached cells were treated with fresh FBS-free medium containing specific concentrations of triptolide. Wound-healing was detected at 0 and 24 h within the scraped lines using an EVOS XL Core cell imaging system. Images were further analyzed using ImageJ (NIH, Bethesda, MD, USA) to calculate the wound-healing rates according to the change in wound width between 0 and 24 h in each group. Three independent experiments were conducted.

### Transwell migration and invasion assays

Cells pretreated with or without triptolide were harvested and suspended in 100 μL of serum-free medium and seeded in the upper compartment of the Transwell insert at a density of 2 × 10^4^ cells/well, while the lower chambers were filled with 600 μL of growth medium. After incubation for another 24 h, the medium within the insert was carefully removed, and cells were fixed with 4% paraformaldehyde for 30 min. Nonmigrated cells on the upper sides of membranes were removed by cotton swabs, and cells on the underside were stained with 0.1% crystal violet for 60 s. For each experiment, the cells in five random fields (magnification, ×10) were counted, and three independent inserts were analyzed.

The Transwell invasion assay was performed using the same method as the Transwell migration assay, except that the Transwell inserts were precoated with Matrigel. The experiment was independently conducted three times.

### Western blotting assay

The cells were collected and lysed in ice-cold RIPA buffer containing 0.1 M PMSF, phosphatase inhibitor, and protease inhibitor cocktail to obtain the total protein. Nuclear and cytoplasmic proteins were extracted using NE-PER nuclear and cytoplasmic extraction reagents following the manufacturer’s protocol. The protein concentration was determined by the BCA protein quantification kit. Equal amounts of protein (30 μg) were separated by 8%–12% SDS-PAGE and transferred onto polyvinylidene difluoride membranes (Millipore, Bedford, MA, USA). Then, the membranes were probed with primary antibodies at 4 °C overnight, followed by incubation with HRP-conjugated secondary antibodies at room temperature for 90 min. The blots were visualized with an enhanced chemiluminescence kit (Millipore) and captured using an Amersham Imager 600 (GE, Boston, USA). Quantitative data were acquired using Image-Pro Plus 6.0 software (NIH, NY, USA).

### Quantitative real-time reverse transcriptase-polymerase chain reaction (RT-qPCR)

Total RNA was extracted using TRIzol (Invitrogen) according to the manufacturer’s protocol. cDNA was prepared with a reverse transcription kit using a LightCycler^®^480 system (Roche, Switzerland). RT-qPCR was performed using the One-Step SYBR PrimeScript Plus RT-PCR kit. The primers presented in Supplementary Table S1 were synthesized by Qingke Biotechnology (Beijing, China). Briefly, primers, TB Green™ Premix Ex Taq™ II and cDNA templates were mixed for the PCR reactions. RT-qPCR analysis was performed in a LightCycler^®^480 Real-Time PCR System (Roche, Switzerland) with the following cycling parameters: 45 cycles of 95 °C for 10 s, 60 °C for 20 s, and 72 °C for 20 s. Relative mRNA expression levels of target genes were standardized with the housekeeping gene GAPDH, the value of which was set as 1. The fold change of mRNA was calculated using the 2^−ΔΔCT^ method.

### Immunofluorescence assay

After treatment with or without triptolide for 24 h, the cells were stained using 4% paraformaldehyde for 15 min, permeated with 0.5% Triton X-100 for 15 min, and blocked using 5% BSA for 1 h. Next, the cells were incubated with primary antibodies (1:200 dilutions) overnight at 4 °C, followed by staining with Alexa Fluor 594 donkey anti-rabbit IgG (1:2000) for 1 h at room temperature. DAPI was used to recognize the nucleus. F-actin was visualized by incubation with phalloidin-Atto 565 (50 μg/mL) for 30 min. Images were taken using a laser scanning confocal microscope (LSM 800, Zeiss) with a ×63 objective.

### Transient transfection

NCI-H1299 or NCI-H460 cells were seeded in six-well plates and grown to 50%–70% confluence. The pCMV3-CTNNB1-EGFP and pCMV3-EGFP vectors were transfected into NCI-H1299 or NCI-H460 cells using Lipofectamine LTX&PLUS reagent following the manufacturer’s protocol. Western blotting and RT-qPCR assays were performed to detect the transfection efficacy after 48 h. The transfected cells were used for transwell migration and invasion assays after 48 h of transfection.

### RNA interference

Nonspecific and β-catenin siRNAs were designed and synthesized by Qingke Biotechnology (Beijing, China). Briefly, NCI-H1299 or NCI-H460 cells were seeded in six-well plates and grown to 30%–50% confluence, and siRNA transfection was performed by using Lipofectamine 3000 according to the manufacturer’s instructions. β-Catenin was knocked down by using the following siRNA duplexes: 5′-GUCAACGUCUUGUUCAGAATT-3′ and 5′-UUCUGAACAAGACGUUGACTT-3′. The negative siRNA duplex that did not target any gene product was used as a negative control, the sequences of which were 5′-UUCUCCGAACGUGUCACGUTT-3′ and 5′-ACGUGACACGUUCGGAGAATT-3′. Transfection efficacy was determined via RT-qPCR and Western blotting assays after 24 h transfection.

### Acute toxicity study in mice

The mice were divided into six groups with ten mice per group. Triptolide was aseptically suspended in 0.9% NaCl solution. The mice were intraperitoneally injected with 3, 3.5, 4, 4.5, 5, and 6 mg/kg triptolide. The toxic symptoms of mice were observed for a period of 48 h. All deaths were recorded. The LD_50_ values and 95%confidence intervals were obtained using the formulae according to the modified Karber method, LD_50_ = lg^−1^ {*X*_m_ − i ($\mathop {\sum }\nolimits {\mathrm{p}}$ − 0.5)}, S_Lg50_ = $\root {{\mathrm{i}}} \of {{(\mathop {\sum }\nolimits {\mathrm{p}}-\mathop {\sum }\nolimits {\mathrm{p}}2)/({n}-1)}}$, *X* = lg^−1^ (lgLD_50_ ± 1.96 ∗ S_Lg50_).

### Subcutaneous xenograft mouse model

NCI-H1299 cells (2 × 10^6^ cells/per mouse) were suspended in 150 μL of PBS and Matrigel mixture and then subcutaneously implanted into the right armpits of nude mice. When the tumor volume grew to ~50 mm^3^, the mice were randomly assigned to three groups (six mice per group). The mice were intraperitoneally injected with saline, 0.75 mg/kg triptolide or 1.5 mg/kg triptolide every 2 days. The tumor size was measured every other day using a Vernier caliper, and the tumor volume was calculated using the following formula: 0.5 × *a* × *b*^2^, for which *a* refers to the longest diameter and *b* refers to the shortest diameter. Body weight was also measured every 2 days. At the end of the experiment, the mice were euthanized and anatomized. The tumors and organs were harvested and fixed in 4% paraformaldehyde for histological and immunochemical analyses.

### Mouse model of lung metastasis

NCI-H1299 cells (4 × 10^6^) were suspended in 200 μL of PBS and injected into the tail veins of each mouse. The mice were randomly divided into the vehicle and triptolide groups (eight mice per group) and intraperitoneally injected with saline or 1.5 mg/kg triptolide three times per week for a total of six weeks. Finally, the mice were euthanized, and lung tissues were harvested and fixed in 4% paraformaldehyde for further histological and immunochemical investigation.

### Hematoxylin and eosin (H&E) staining and immunohistochemistry (IHC) assay

The tumor and other tissues were embedded in paraffin, sliced into 4-µm-thick sections and mounted on slides. For H&E staining, the sections were stained with H&E according to the manufacturer’s instructions.

For the IHC assay, after antigen retrieval by heating in a microwave, the sections were blocked with 5% BSA and incubated with primary antibodies overnight at 4 °C. The sections were then incubated with biotinylated secondary antibody for 1 h at room temperature, followed by HRP-streptavidin treatment for 15 min. The sections were further stained with 3,3′-diaminobenzidine and counterstained with hematoxylin. Five random fields were captured on each section by two independent investigators.

### Statistical analysis

The data analyses were performed using GraphPad Prism 5.0 (GraphPad Software, Inc., San Diego, CA, USA) and are expressed as the mean ± SEM. The significance of differences among groups was determined by one-way analysis of variance followed by Tukey’s test. In addition, *P* < 0.05 indicated a significant difference.

## Results

### Triptolide attenuates the migration and invasion of NCI-H1299 cells

We first evaluated the effect of triptolide on the viability of NCI-H1299 cells using a CCK-8 assay. The results showed that triptolide substantially decreased the viability of NCI-H1299 cells in a dose-dependent manner, and triptolide did not induce apparent toxicity in NCI-H1299 cells at a concentration of 4.22 nM (Fig. 1b). Three concentrations lower than 4.22 nM (0.5, 1.0, and 2.0 nM) were selected for further in vitro experiments. Next, we conducted a scratch wound-healing assay to determine whether triptolide affects the migratory capacity of NCI-H1299 cells. The results showed that triptolide treatment significantly inhibited wound closure in a dose-dependent manner. Most of the cells migrated to the scraped lines in the vehicle group, whereas the number of migratory cells in the triptolide-treated group was obviously decreased, with only ~70%, 45%, and 30% of the cells migrating to the scraped lines in the 0.5, 1.0, and 2.0 nM groups, respectively (Fig. 1c, d). In the Transwell migration assay, triptolide significantly suppressed the horizontal migratory ability of NCI-H1299 cells, as the number of migrated cells was much lower in the triptolide group than in the vehicle group. Similar results were observed in the Transwell invasion assay, and the invaded cells were notably decreased in the triptolide-treated groups compared with the vehicle group (Fig. 1e–g). The above results suggested that triptolide suppressed the migration and invasion capabilities of NCI-H1299 cells.

### Triptolide suppresses EMT in NCI-H1299 cells

**Fig. 2:**
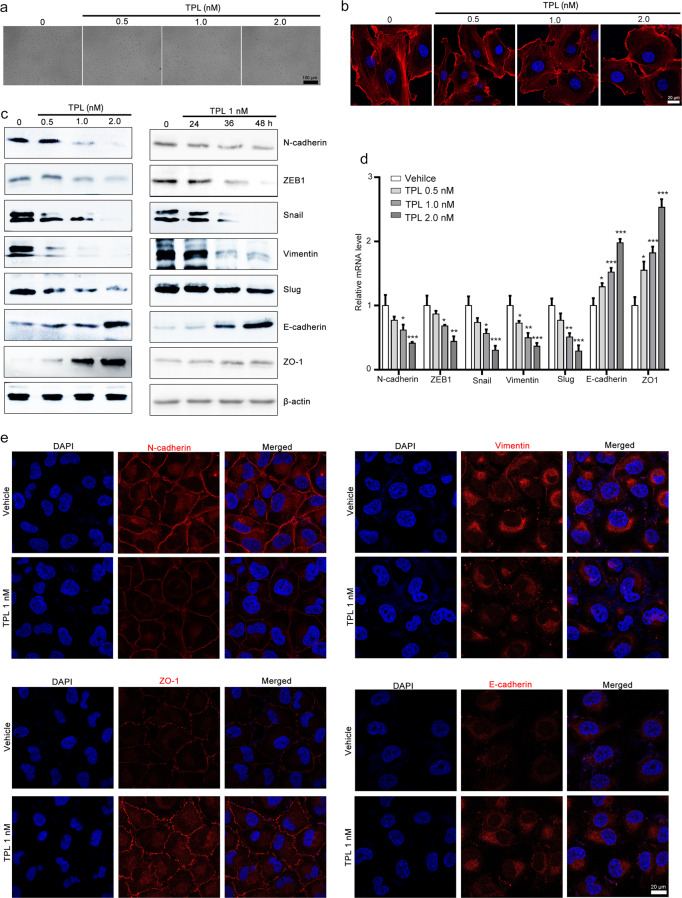
Triptolide suppresses EMT in NCI-H1299 cells. The effect of triptolide on the morphology of NCI-H1299 cells. NCI-H1299 cells were exposed to various concentrations of triptolide for 48 h, and morphological changes were observed with a microscope (×100 magnification) (**a**) and with a laser scanning confocal microscope (×40 magnification) after staining using rhodamine-phalloidin (**b**). **c**, **d** The effect of triptolide on EMT markers in NCI-H1299 cells. The cells were exposed to various concentrations of triptolide for 48 h or 1 nM triptolide for 24, 36, and 48 h. The cells were harvested and subjected to Western blotting and RT-qPCR assays to detect the expression of ZEB1, N-cadherin, vimentin, slug, snail, ZO-1, and E-cadherin. β-Actin and GAPDH were used as internal controls in Western blotting and RT-qPCR assays. The results of Western blotting and RT-qPCR assays are presented in **c** and **d**, respectively. The data are presented as the mean ± SEM, *n* = 3. *P < 0.05, ***P < 0.01 and ***P < 0.001 vs. the vehicle group. **e** Triptolide modulated the expression of EMT markers, as indicated by immunofluorescence staining. NCI-H1299 cells were treated with or without triptolide for 48 h, and then, E-cadherin, ZO-1, vimentin, and ZEB1 expression was detected using an immunofluorescence staining assay. DAPI was used to visualize the nuclei. Images were taken using a Zeiss LSM 800 confocal microscope with a ×63 oil immersion lens. TPL triptolide.

Given that EMT plays a vital role in tumor metastasis, we further investigated whether triptolide affects the EMT of NCI-H1299 cells. Microscopic analyses revealed that triptolide treatment resulted in a morphological change in NCI-H1299 cells from an elongated mesenchymal to a cobblestone-shaped epithelial phenotype (Fig. 2a). We also observed that F-actin filaments were scattered throughout the entire cytoplasm in the vehicle group but were rearranged in the triptolide-treated group (Fig. 2b). Western blotting and RT-qPCR assays showed that triptolide downregulated the expression of mesenchymal markers (including N-cadherin, ZEB1, snail, vimentin, and slug) and upregulated the expression of the epithelial markers E-cadherin and ZO-1 (Fig. 2c). Immunofluorescence assays further confirmed that triptolide treatment significantly increased E-cadherin and ZO-1 expression and decreased N-cadherin and vimentin expression (Fig. 2d). All these results indicated that triptolide suppressed EMT in NCI-H1299 cells.

### Triptolide suppresses β-catenin expression in NCI-H1299 and NCI-H460 cells

**Fig. 3:**
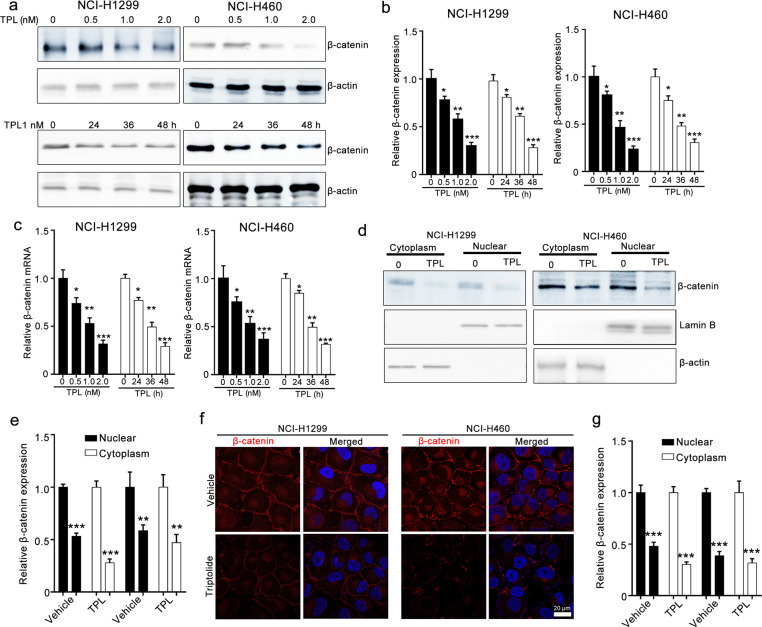
Triptolide reduces β-catenin expression in NCI-H1299 and NCI-H460 cells. **a**–**c** The effect of triptolide on the protein and mRNA levels of β-catenin in NCI-H1299 and NCI-H460 cells. The cells were treated with triptolide for the indicated times and then collected for Western blotting assays and RT-qPCR assays. Representative images and quantitative Western blotting data are presented in **a** and **b**. The RT-qPCR results are shown in **c**. **d**, **e** The effect of triptolide on the nuclear translocation of β-catenin. The cells were lysed by using a NE-PERTM Nuclear and Cytoplasmic Extraction Kit and subjected to Western blotting assays. β-Actin and Lamin B were used as loading controls. Representative images and quantitative data are shown in **d** and **e**, respectively. **f**, **g** The effect of triptolide on β-catenin translocation from the cytoplasm to the nucleus, as indicated by immunofluorescence assays. After treatment with triptolide for 24 h, the cells were fixed with 4% paraformaldehyde, blocked using 5% BSA, and incubated with β-catenin antibody, followed by Alexa Fluor 594-conjugated donkey anti-rabbit IgG and DAPI. TPL triptolide. Representative images and quantitative data are shown in **f** and **g**, respectively. Images were taken using a Zeiss LSM 800 confocal microscope with a 63× oil immersion lens. TPL triptolide. Quantitative data are presented as the mean ± SEM, *n* = 3. *P < 0.05, **P < 0.01 and ***P < 0.001 vs. the vehicle group.

As β-catenin is a crucial regulator of EMT [27], we assessed whether triptolide suppressed EMT by modulating β-catenin. Western blotting assays showed that triptolide treatment significantly decreased β-catenin expression in a dose- and time-dependent manner in NCI-H1299 and NCI-H460 cells (Fig. 3a–c). We further collected subcellular protein fractions of NCI-H1299 and NCI-H460 cells and estimated the β-catenin level in the cytoplasm and nucleus using Western blotting assays. The results revealed that triptolide reduced β-catenin expression in both the nucleus and cytoplasm (Fig. 3d, e). Immunofluorescence assays also showed that triptolide decreased the β-catenin levels in both the nucleus and cytoplasm of NCI-H1299 and NCI-H460 cells (Fig. 3f, g). These data indicated that β-catenin might be involved in triptolide-mediated EMT.

### Triptolide inhibits EMT by downregulating β-catenin expression

**Fig. 4:**
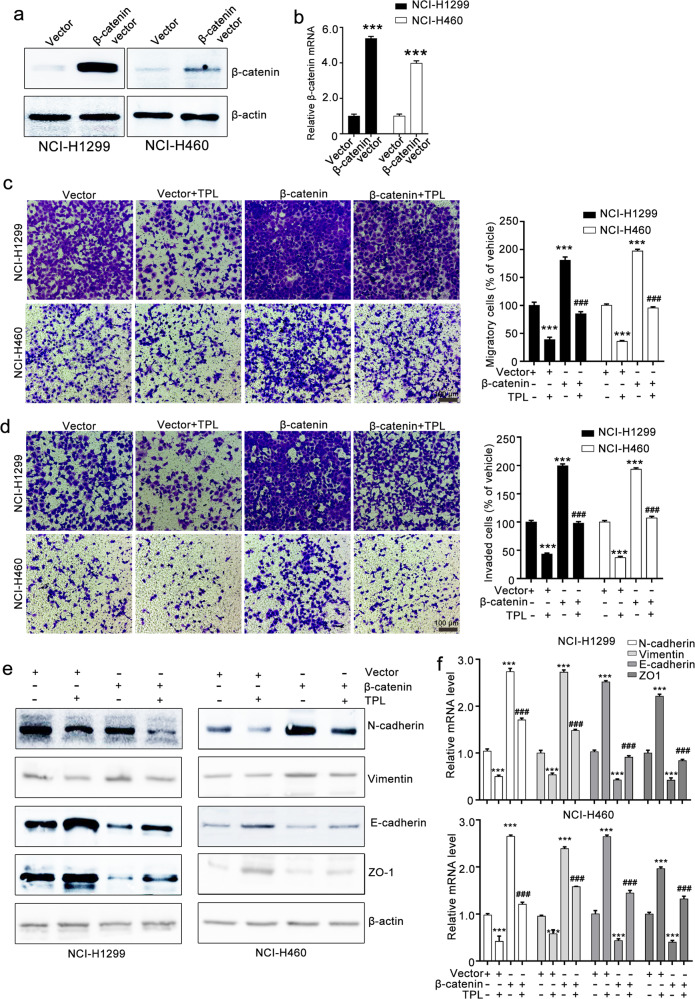
β-Catenin overexpression attenuates the triptolide-induced inhibitory effect on NCI-H1299 and NCI-H460 cells. **a**, **b** β-Catenin vector transfection increased β-catenin expression, as detected by Western blotting and RT-qPCR assays. **c**, **d** The effects of β-catenin overexpression on the triptolide-mediated inhibitory effect on the migration and invasion of NCI-H1299 and NCI-H460 cells. NCI-H1299 and NCI-H460 cells were transfected with NC vector or β-catenin vector and then subjected to Transwell migration assays (**c**) and invasion assays (**d**). The images were taken at ×100 magnification. **e**, **f** The effect of triptolide on EMT in NCI-H1299 and NCI-H460 cells was weakened by β-catenin overexpression. After transfection, the cells were collected and used for Western blotting and RT-qPCR assays. The results of Western blotting and RT-qPCR assays are shown in **e** and **f**, respectively. TPL triptolide. The data are presented as the mean ± SEM, *n* = 3. ***P < 0.001 compared with the vector group, ^###^*P* < 0.001 vs. the β-catenin vector group.

**Fig. 5:**
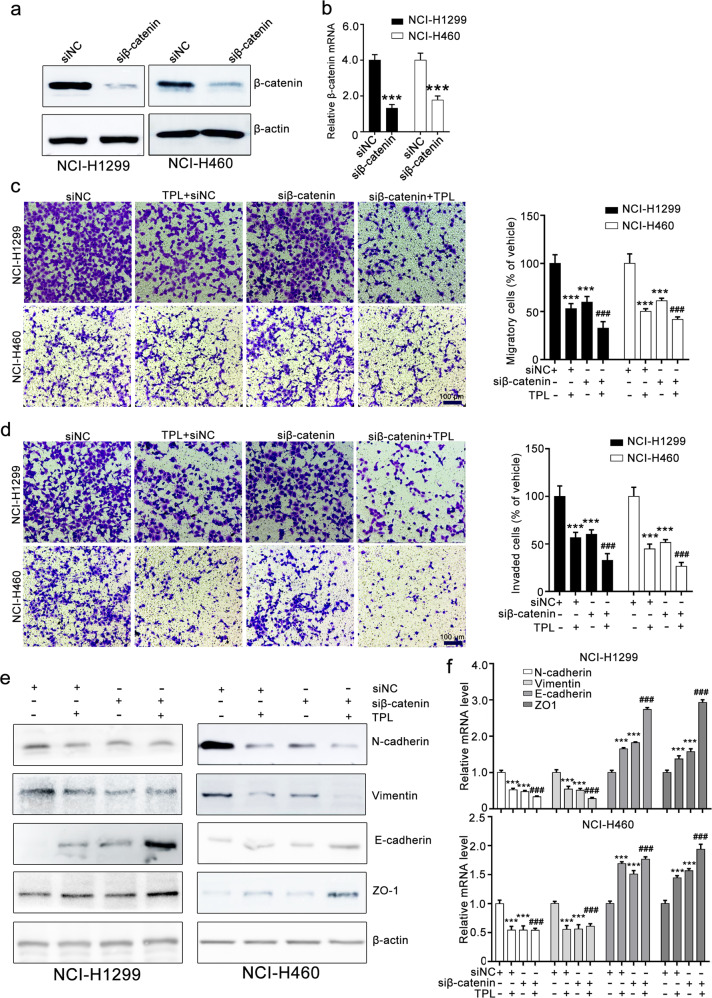
β-Catenin siRNA mildly enhances triptolide-induced inhibition of NCI-H1299 and NCI-H460 cells. **a**, **b** β-Catenin siRNA transfection decreased β-catenin expression, as measured by Western blotting and RT-qPCR assays. **c**, **d** The effect of triptolide combined with β-catenin siRNA on the migration and invasion of NCI-H1299 and NCI-H460 cells as indicated by Transwell migration and invasion assays. The images were taken at ×100 magnification. **e**, **f** The effect of triptolide combined with β-catenin siRNA on EMT as indicated by Western blotting and RT-qPCR assays. TPL triptolide. The data are presented as the mean ± SEM, *n* = 3. ***P < 0.001 vs. the siNC group, ^###^*P*< 0.001 vs. the β-catenin vector group.

To further investigate whether the triptolide-mediated inhibitory effect on EMT is attributed to the suppression of β-catenin, we constructed β-catenin-overexpressing cells. Western blotting and RT-qPCR assays confirmed that the β-catenin vector strongly increased β-catenin expression in NCI-H1299 and NCI-H460 cells (Fig. 4a, b). Transwell migration and invasion assays showed that β-catenin overexpression attenuated triptolide-induced inhibition of the migration and invasion of NCI-H1299 and NCI-H460 cells. Triptolide treatment suppressed the migratory and invasive ability of NCI-H1299 and NCI-H460 cells, whereas β-catenin overexpression counteracted the triptolide-mediated inhibitory effect on the migration and invasion of NCI-H1299 and NCI-H460 cells (Fig. 4c, d). Western blotting assays revealed that β-catenin overexpression antagonized the triptolide-mediated inhibitory effect on EMT marker expression (Fig. 4e, f). We also silenced β-catenin expression using siRNA and confirmed the transfection efficacy with Western blotting and RT-qPCR assays (Fig. 5a, b). Further Transwell migration and invasion assays showed that β-catenin siRNA enhanced the triptolide-induced inhibitory effect on the migration and invasion of NCI-H1299 and NCI-H460 cells (Fig. 5c, d). β-Catenin siRNA transfection suppressed the expression of EMT markers, and this effect was augmented by triptolide treatment (Fig. 5e, f). These results revealed that triptolide suppressed the migration, invasion, and EMT of NCI-H1299 and NCI-H460 cells by downregulating β-catenin expression.

### Triptolide suppresses EMT in a xenograft model and reduces lung metastasis in a tail vein injection model

**Fig. 6:**
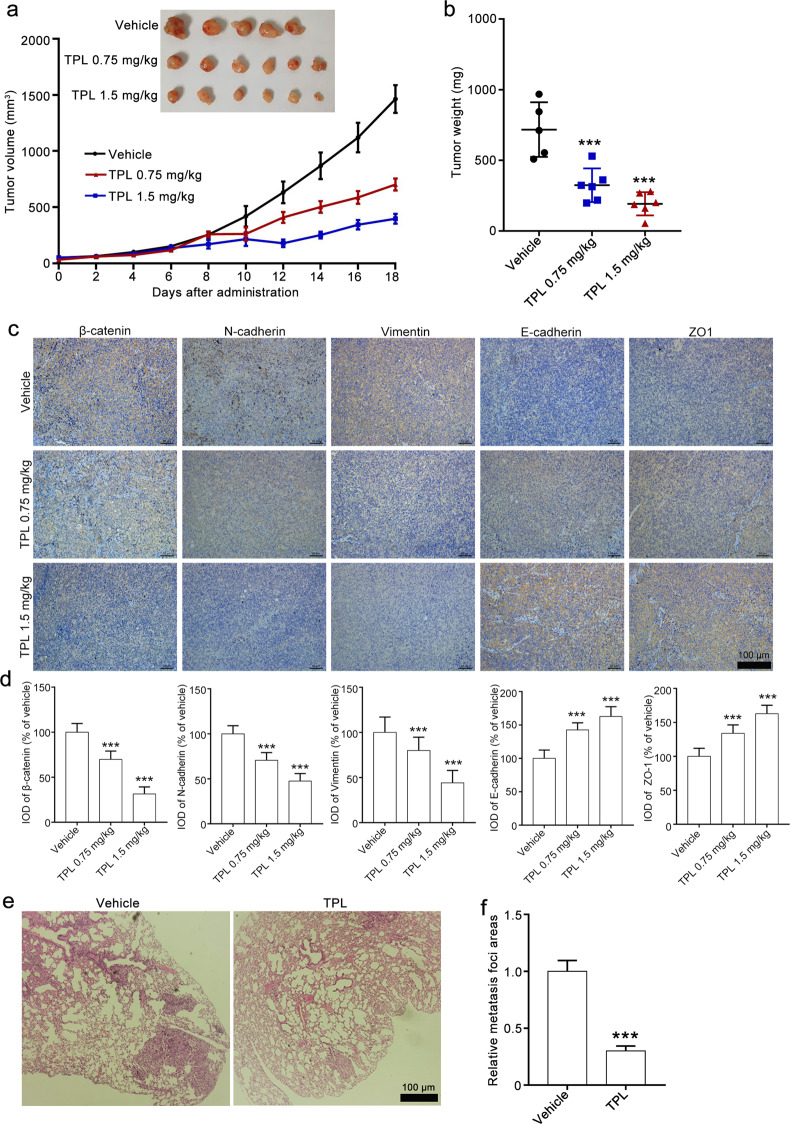
Triptolide suppresses tumor growth in a xenograft model and lung metastasis of NCI-H1299 cells. **a**, **b** The antitumor effect of triptolide on the NCI-H1299 xenograft model. Tumor-bearing mice were administered vehicle and triptolide (1.5 and 0.75 mg/kg) every 2 days for a total of 18 days. At the end of the experiment, the mice were sacrificed, and the tumors were harvested. The tumor volume curve and tumor weight are presented in **a** and **b**, respectively. **c**, **d** The effect of triptolide on the expression of β-catenin and EMT markers in NCI-H1299 xenograft tumors. Immunohistochemical assays were conducted to detect β-catenin, N-cadherin, vimentin, E-cadherin, and ZO-1 expression. Representative images and quantitative data are presented in **c** and **d**. The images were taken at ×100 magnification. **e**, **f** The effect of triptolide on the lung metastasis of NCI-H1299 cells. After tail vein injections of NCI-H1299 cells, the mice were administered triptolide (1.5 mg/kg) three times per week for a total of 6 weeks. H&E staining of lung sections and quantitative data of metastatic foci are shown in **e** and **f**. H&E images were taken at ×100 magnification. TPL triptolide. The data are presented as the mean ± SEM, *n* = 5 in vehicle group. *n* = 6 in TPL group.  ***P < 0.001 vs. the vehicle group.

Next, we used an NCI-H1299 xenograft tumor model to assess whether triptolide suppresses EMT in vivo. Triptolide (1.5 and 0.75 mg/kg) administration resulted in obvious reductions in tumor volume and weight compared with that of the vehicle group (Fig. 6a, b). We did not observe a significant body weight difference between the triptolide group and the vehicle group (Supplementary Fig. S1a). The results of H&E staining showed that triptolide treatment did not lead to significant histological alterations in the heart, liver, spleen, lungs, or kidneys of the mice (Supplementary Fig. S1b). We also conducted an acute toxicity study of triptolide and found that the LD_50_ value of triptolide in mice was 3.22 mg/kg (Supplementary Table S2). All these results suggest that triptolide at doses of 1.5 and 0.75 mg/kg may have low toxicity in tumor-bearing mice.

The results of IHC staining demonstrated that triptolide treatment reduced the β-catenin levels. We also observed that triptolide treatment led to downregulation of N-cadherin and vimentin expression and upregulation of E-cadherin and ZO-1 expression (Fig. 6c, d). We then developed a pulmonary metastasis model by tail vein injection of tumor cells to assess the effect of triptolide on lung metastasis. The results showed that circulating NCI-H1299 cells accumulated in the lung and developed into metastatic foci in the vehicle group, and triptolide (1.5 mg/kg) administration substantially suppressed the lung metastasis induced by NCI-H1299 cells, as the number of metastatic foci was less than half of that in the vehicle group (Fig. 6e, f). These results indicated that triptolide suppressed the metastasis of NCI-H1299 cells in vivo by reversing β-catenin-mediated EMT.

## Discussion

Metastasis is the major cause leading to the therapeutic failure and mortality of NSCLC. Although a number of specific and effective agents or therapeutic strategies for treating NSCLC have been developed and applied, the 5-year survival of NSCLC patients is still unsatisfactory [4]. EMT is critical for NSCLC metastasis, and targeting EMT has been recognized as a promising therapeutic strategy for metastatic NSCLC patients [28]. In the present study, our results showed that triptolide suppressed the migration and invasion of NSCLC cells by reversing EMT. Moreover, triptolide suppressed EMT in NCI-H1299 xenograft tumors and decreased lung metastasis in a tail vein injection model without significant toxicity in major organs at doses of 1.5 and 0.75 mg/kg. Further investigation revealed that the triptolide-induced inhibitory effect on EMT and metastasis was partially attributed to the suppressive effect on β-catenin expression. Our study provides strong evidence that triptolide is a potential agent for the treatment of metastatic NSCLC.

Triptolide, a major active component from *T. wilfordii* Hook F, shows antitumor effects in many cancers, such as breast cancer, pancreatic AC, and lung cancer, by suppressing cell proliferation, inducing cell apoptosis, and reversing stem-like features by regulating the HSP70, NF-κB, and p53 pathways [20, 29, 30]. Recently, several studies have indicated that inhibition of metastasis was involved in the anticancer activity of triptolide, but systematic evaluations were lacking, and the underlying mechanism was still unclear [26, 30, 31]. In the present study, we systematically evaluated the effect of triptolide on NSCLC metastasis in vitro and in vivo using wound-healing assays, Transwell migration and invasion assays, subcutaneous xenograft mouse models and mouse models of lung metastasis. Our results showed that triptolide downregulated β-catenin expression in NSCLC cells by promoting its phosphorylation and degradation, thus suppressing EMT and cell motilities (migration and invasion) (Supplementary Fig. S2). These results suggest the potential of triptolide for the treatment of metastatic NSCLC. Moreover, a previous study showed that EMT was correlated with chemoresistance [32], and triptolide could sensitize several cancer cell lines to cisplatin, 5-FU or carboplatin in vitro and in vivo [33]. Considering that triptolide reversed EMT, we speculate that triptolide could be used in combination with other chemotherapeutics to achieve a synergistic effect on NSCLC. In this regard, our results also suggest that triptolide may enhance chemotherapeutic efficacy to inhibit metastatic recurrence of NSCLC and provide new clues for further exploration of the potencies of triptolide for the treatment of metastatic NSCLC, which will be examined in our future studies.

EMT is crucial for the metastasis of various cancers, including breast cancer, pancreatic cancer, colorectal cancer, and NSCLC. EMT is a process characterized by elevated expression of mesenchymal markers (vimentin and N-cadherin) and loss of the expression of an epithelial marker (E-cadherin), which is regulated by a complex network of signaling pathways and transcription factors [34, 35]. Many studies have shown that EMT is crucial for NSCLC metastasis. Soluble E-cadherin has been employed as one of the markers of EMT and an indicator of NSCLC patient survival. Suppressing or reversing EMT with curcumin or resveratrol could reduce NSCLC metastasis, indicating that targeting EMT is an effective therapeutic strategy for metastatic NSCLC [36]. However, the role of EMT in NSCLC metastasis and the underlying mechanism have not been extensively characterized [37, 38]. Thus, revealing the molecular mechanism regulating the EMT process in NSCLC is vital for developing therapeutic strategies for NSCLC metastasis. In this study, we showed that β-catenin was critical for the EMT of NSCLC. β-Catenin overexpression increased the expression of mesenchymal markers (N-cadherin and vimentin), reduced the expression of epithelial markers (E-cadherin and ZO-1), and attenuated the triptolide-mediated inhibitory effect on the migration, invasion, and EMT of NCI-H1299 and NCI-H460 cells, whereas β-catenin knockdown enhanced these effects of triptolide. These results suggest that β-catenin is crucial for the EMT and metastasis of NSCLC and that targeting β-catenin is a potential therapeutic strategy for metastatic NSCLC.

β-Catenin, identified as a transcriptional regulator, plays a critical role in canonical Wnt signaling. Membrane β-catenin binds to the cytoplasmic region of E-cadherin to form a complex and then promotes cell adhesion [39]. When stimulated with Wnt or genetic mutations of Wnt components, β-catenin accumulates in the cytoplasm, translocates into the nucleus and binds to LEF-1/TCF4 and some other coregulators, leading to the expression of target genes, such as c-Myc and MMP7. Nuclear β-catenin accumulation indicates poor survival and has been recognized as an independent prognostic factor in NSCLC patients [40, 41]. Moreover, β-catenin has been identified as a vital regulator of EMT-mediated tumor metastasis. Several EMT markers, including E-cadherin, vimentin, ZEB1, and slug, are directly or indirectly regulated by β-catenin [42, 43]. In the present study, we found that β-catenin was critical for the triptolide-mediated suppressive effect on the EMT and metastasis of NSCLC. GSK3β is a critical regulator of β-catenin destabilization, which promotes the phosphorylation and ubiquitination of β-catenin, leading to β-catenin proteasomal degradation [44]. Thus, we detected the effect of triptolide on p-β-catenin and p-GSK3β^ser9^ expression. The results showed that triptolide treatment promoted β-catenin phosphorylation and reduced p-GSK3β^ser9^ expression (Supplementary Fig. S2a), indicating that triptolide may promote GSK3β-mediated β-catenin degradation. We stimulated the cells with Wnt3a to activate the Wnt/β-catenin signaling pathway and found that triptolide mimicked Wnt3a-induced β-catenin upregulation (Supplementary Fig. S2b). We also pretreated the cells with LiCl and MG132 to block GSK3β activation and suppress β-catenin degradation and found that the triptolide-mediated inhibitory effect on β-catenin expression was abolished (Supplementary Fig. S2c, d). All these results indicated that there was no crosstalk between the triptolide-induced inhibitory effect on β-catenin expression and the Wnt/β-catenin pathway in NCI-H1299 cells. Triptolide may downregulate β-catenin expression by inducing GSK3β-mediated β-catenin degradation. We will investigate the mechanism underlying triptolide-mediated β-catenin degradation in future studies.

In summary, our study demonstrated that triptolide inhibited EMT and metastasis of NSCLC partially through downregulation of β-catenin. Our study suggests that β-catenin could be considered a therapeutic target for metastatic NSCLC and that triptolide may serve as a candidate drug for metastatic NSCLC.

## Supplementary information

Supplementary Information

Supplementary Figure 1

Supplementary Figure 2

## References

[1] Siegel RL, Miller KD, Jemal A (2020). Cancer statistics, 2020. CA Cancer J Clin.

[2] Herbst RS, Morgensztern D, Boshoff C (2018). The biology and management of non-small cell lung cancer. Nature..

[3] Feng RM, Zong YN, Cao SM, Xu RH (2019). Current cancer situation in China: good or bad news from the 2018 Global Cancer Statistics?. Cancer Commun.

[4] Hirsch FR, Scagliotti GV, Mulshine JL, Kwon R, Curran WJ, Curran WJ (2017). Lung cancer: current therapies and new targeted treatments. Lancet..

[5] Grzeskowiak CL, Kundu ST, Mo X, Ivanov AA, Zagorodna O, Lu H (2018). In vivo screening identifies GATAD2B as a metastasis driver in KRAS-driven lung cancer. Nat Commun.

[6] Neelakantan D, Zhou H, Oliphant MUJ, Zhang X, Simon LM, Henke DM (2017). EMT cells increase breast cancer metastasis via paracrine GLI activation in neighbouring tumour cells. Nat Commun.

[7] Lamouille S, Xu J, Derynck R (2014). Molecular mechanisms of epithelial-mesenchymal transition. Nat Rev Mol Cell Biol.

[8] Yang S, Liu Y, Li MY, Ng CSH, Yang SL, Wang S (2017). FOXP3 promotes tumor growth and metastasis by activating Wnt/β-catenin signaling pathway and EMT in non-small cell lung cancer. Mol Cancer.

[9] Manshouri R, Coyaud E, Kundu ST, Peng DH, Stratton SA, Alton K (2019). ZEB1/NuRD complex suppresses TBC1D2b to stimulate E-cadherin internalization and promote metastasis in lung cancer. Nat Commun.

[10] Matsubara D, Kishaba Y, Yoshimoto T, Sakuma Y, Sakatani T, Tamura T (2014). Immunohistochemical analysis of the expression of E-cadherin and ZEB1 in non-small cell lung cancer. Pathol Int.

[11] Larsen JE, Nathan V, Osborne JK, Farrow RK, Deb D, Sullivan JP (2016). ZEB1 drives epithelial-to-mesenchymal transition in lung cancer. J Clin Invest.

[12] Liu X, Li C, Yang Y, Liu X, Li R, Zhang M (2019). Synaptotagmin 7 in twist-related protein 1-mediated epithelial-mesenchymal transition of non-small cell lung cancer. EBioMedicine..

[13] Raungrut P, Wongkotsila A, Champoochana N, Lirdprapamongkol K, Svasti J, Thongsuksai P (2018). Knockdown of 14-3-3γ suppresses epithelial-mesenchymal transition and reduces metastatic potential of human non-small cell lung cancer cells. Anticancer Res.

[14] Treesuwan S, Sritularak B, Chanvorachote P, Pongrakhananon V (2018). Cypripedin diminishes an epithelial-to-mesenchymal transition in non-small cell lung cancer cells through suppression of Akt/GSK-3β signalling. Sci Rep.

[15] Kapinova A, Stefanicka P, Kubatka P, Zubor P, Uramova S, Kello M (2017). Are plant-based functional foods better choice against cancer than single phytochemicals? A critical review of current breast cancer research. Biomed Pharmacother.

[16] Zhang XW, Liu W, Jiang HL, Mao B (2018). Chinese herbal medicine for advanced non-small-cell lung cancer: A systematic review and meta-analysis. Am J Chin Med.

[17] Reno TA, Tong SW, Wu J, Fidler JM, Nelson R, Kim JY (2016). The triptolide derivative MRx102 inhibits Wnt pathway activation and has potent anti-tumor effects in lung cancer. BMC Cancer.

[18] Liu Q (2011). Triptolide and its expanding multiple pharmacological functions. Int Immunopharmacol.

[19] Ma Y, Wang Y, Wang L, Qian Y, Jian Y, Kong P (2019). Triptolide prevents proliferation and migration of esophageal squamous cell cancer via MAPK/ERK signaling pathway. Eur J Pharmacol.

[20] Kim ST, Kim SY, Lee J, Kim K, Park SH, Park YS (2018). Triptolide as a novel agent in pancreatic cancer: the validation using patient derived pancreatic tumor cell line. BMC Cancer.

[21] Noel P, Von Hoff DD, Saluja AK, Velagapudi M, Borazanci E, Han H (2019). Triptolide and its derivatives as cancer therapies. Trends Pharmacol Sci.

[22] Meng C, Zhu H, Song H, Wang Z, Huang G, Li D (2014). Targets and molecular mechanisms of triptolide in cancer therapy. Chin J Cancer Res.

[23] Sarkar TR, Battula VL, Werden SJ, Vijay GV, Ramirez-Pena EQ, Taube JH (2015). GD3 synthase regulates epithelial-mesenchymal transition and metastasis in breast cancer. Oncogene.

[24] Zhao H, Yang Z, Wang X, Zhang X, Wang M, Wang Y (2012). Triptolide inhibits ovarian cancer cell invasion by repression of matrix metalloproteinase 7 and 19 and upregulation of E-cadherin. Exp Mol Med.

[25] Nomura A, Majumder K, Giri B, Dauer P, Dudeja V, Roy S (2016). Inhibition of NF-kappa B pathway leads to deregulation of epithelial-mesenchymal transition and neural invasion in pancreatic cancer. Lab Invest.

[26] Song JM, Molla K, Anandharaj A, Cornax I, O Sullivan MG, Kirtane AR (2017). Triptolide suppresses the in vitro and in vivo growth of lung cancer cells by targeting hyaluronan-CD44/RHAMM signaling. Oncotarget.

[27] Zhou P, Li Y, Li B, Zhang M, Liu Y, Yao Y (2019). NMIIA promotes tumor growth and metastasis by activating the Wnt/β-catenin signaling pathway and EMT in pancreatic cancer. Oncogene..

[28] Mittal V (2016). Epithelial mesenchymal transition in aggressive lung cancers. Adv Exp Med Biol.

[29] Liu L, Salnikov AV, Bauer N, Aleksandrowicz E, Labsch S, Nwaeburu C (2014). Triptolide reverses hypoxia-induced epithelial-mesenchymal transition and stem-like features in pancreatic cancer by NF-κB downregulation. Int J Cancer.

[30] Reno TA, Kim JY, Raz DJ (2015). Triptolide inhibits lung cancer cell migration, invasion, and metastasis. Ann Thorac Surg..

[31] Nardi I, Reno T, Yun X, Sztain T, Wang J, Dai H (2018). Triptolide inhibits Wnt signaling in NSCLC through upregulation of multiple Wnt inhibitory factors via epigenetic modifications to Histone H3. Int J Cancer.

[32] Fukuda K, Takeuchi S, Arai S, Katayama R, Nanjo S, Tanimoto A (2019). Epithelial-to-mesenchymal transition is a mechanism of ALK inhibitor resistance in lung cancer independent of ALK mutation status. Cancer Res.

[33] Zhu J, Wang H, Chen F, Lv H, Xu Z, Fu J (2018). Triptolide enhances chemotherapeutic efficacy of antitumor drugs in non-small-cell lung cancer cells by inhibiting Nrf2-ARE activity. Toxicol Appl Pharmacol.

[34] Pastushenko I, Blanpain C (2019). EMT transition states during tumor progression and metastasis. Trends Cell Biol.

[35] Bakir B, Chiarella AM, Pitarresi JR, Rustgi AK (2020). EMT, MET, plasticity and tumor metastasis. Trends Cell Biol.

[36] Loh CY, Chai JY, Tang TF, Wong WF, Sethi G, Shanmugam MK (2019). The E-cadherin and N-cadherin switch in epithelial-to-mesenchymal transition: signaling, therapeutic implications, and challenges. Cells..

[37] Tsoukalas N, Aravantinou-Fatorou E, Tolia M, Giaginis C, Galanopoulos M, Kiakou M (2017). Epithelial-mesenchymal transition in non small-cell lung cancer. Anticancer Res.

[38] Reka AK, Chen G, Jones RC, Amunugama R, Kim S, Karnovsky A (2014). Epithelial-mesenchymal transition-associated secretory phenotype predicts survival in lung cancer patients. Carcinogenesis..

[39] Vishnoi K, Viswakarma N, Rana A, Rana B (2020). Transcription factors in cancer development and therapy. Cancers..

[40] Li XQ, Yang XL, Zhang G, Wu SP, Deng XB, Xiao SJ (2013). Nuclear β-catenin accumulation is associated with increased expression of Nanog protein and predicts poor prognosis of non-small cell lung cancer. J Transl Med.

[41] Chiu CG, Chan SK, Fang ZA, Masoudi H, Wood-Baker R, Jones SJM (2012). Beta-catenin expression is prognostic of improved non-small cell lung cancer survival. Am J Sur.

[42] Sánchez-Tilló E, de Barrios O, Siles L, Cuatrecasas M, Castells A, Postigo A (2011). β-catenin/TCF4 complex induces the epithelial-to-mesenchymal transition (EMT)-activator ZEB1 to regulate tumor invasiveness. Proc Natl Acad Sci USA.

[43] Chen L, Mai W, Chen M, Hu J, Zhuo Z, Lei X (2017). Arenobufagin inhibits prostate cancer epithelial-mesenchymal transition and metastasis by down-regulating β-catenin. Pharmacol Res.

[44] Liu C, Kato Y, Zhang Z, Do VM, Yankner BA, He X (1999). beta-Trcp couples beta-catenin phosphorylation-degradation and regulates Xenopus axis formation. Proc Natl Acad Sci USA.

