# Supplementary material for: Triptolide suppresses the growth and metastasis of non-small cell lung cancer by inhibiting β-catenin-mediated epithelial–mesenchymal transition
Source: Acta Pharmacol Sin. 2021 Apr 23;42(9):1486–97. doi: 10.1038/s41401-021-00657-w (PMC8379262; doi:10.1038/s41401-021-00657-w)
Supplement: Supplementary file 1 — Supplementary Information [file 41401_2021_657_MOESM1_ESM.docx]

**1. Supplementary Tables**

**Supplementary Table S1. Prime sequences used in RT-qPCR assay**

| Gene name | Forward/Reverse | Sequence (5’-3’) |
| --- | --- | --- |
| β-Catenin | Forward  Reverse | GAAACGGCTTTCAGTTGAGC  CTGGCCATATCCACCAGAGT |
| N-cadherin | Forward  Reverse | AGCCAACCTTAACTGAGGAGT  GGCAAGTTGATTGGAGGGATG |
| Vimentin | Forward  Reverse | GCCCTAGACGAACTGGGTC GGCTGCAACTGCCTAATGAG |
| ZEB1 | Forward  Reverse | GCACCTGAAGAGGACCAGAG  GTGTAACTGCACAGGGAGCA |
| Snail | Forward  Reverse | TCGGAAGCCTAACTACAGCGA AGATGAGCATTGGCAGCGAG |
| Slug | Forward  Reverse | CGAACTGGACACACATACAGTG GCCTAGAGTTGCCGACTTATG |
| E-cadherin  ZO1 | Forward  Reverse  Forward  Reverse | CGAGAGCTACACGTTCACGG GGGTGTCGAGGGAAAAATAGG  CCCCACTCTGAAAATGAGGA  GGGAACAACATACAGTGACGC |
| GAPDH | Forward  Reverse | GAAAGCCTGCCGGTGACTAA  TGGAATTTGCCATGGGTGGA |

**Supplementary Table S2. Survival number and LD_50_ of triptolide in acute intraperitoneal toxicity study.**

| Dose (mg/kg) | Total number | Number of deaths | Mortality (%) | LD_50_ (mg/kg) | 95% Credibility |
| --- | --- | --- | --- | --- | --- |
| 3 | 10 | 0 | 0 | 3.22 | 2.58-3.99 |
| 3.5 | 10 | 2 | 0.2 |  |  |
| 4 | 10 | 5 | 0.5 |  |  |
| 4.5 | 10 | 6 | 0.6 |  |  |
| 5 | 10 | 9 | 0.9 |  |  |
| 6 | 10 | 10 | 1.0 |  |  |

**2. Supplementary Figures**

**Supplementary Fig. S1 Triptolide (1.5 and 0.75 mg/kg) has no significant toxicity towards tumor bearing mice. a** Triptolide (1.5 and 0.75 mg/kg) has no significant effect on the body weight of mice. **b** Triptolide has mimic toxic effect on the major organs of mice at the dose of 1.5 and 0.75 mg/kg. The representative images were shown (100 × magnifications).

**Supplementary Fig. S2 Triptolide-mediated inhibitory effect of β-catenin expression is not involved in wnt/β-catenin pathway. a** Triptolide treatment promoted the phosphorylation of β-catenin. **b, c** Triptolide-induced inhibitory effect of β-catenin expression was not depend on wnt/β-catenin pathway. NCI-H1299 cells were treated with 20 mM LiCl (**b**), or 0.4 μM MG132 (**c**) in combination with triptolide for 48 h. And then the cells were applied for western blotting assay to detect β-catenin expression. β-Actin was used as a loading control. The data are shown as mean ± SEM, *n*=3. ^***^*P* < 0.001 vs. the Vehicle group.
