# Supplementary figures and images for: Triptolide suppresses the growth and metastasis of non-small cell lung cancer by inhibiting β-catenin-mediated epithelial–mesenchymal transition
Source: Acta Pharmacol Sin. 2021 Apr 23;42(9):1486–97. doi: 10.1038/s41401-021-00657-w (PMC8379262; doi:10.1038/s41401-021-00657-w)

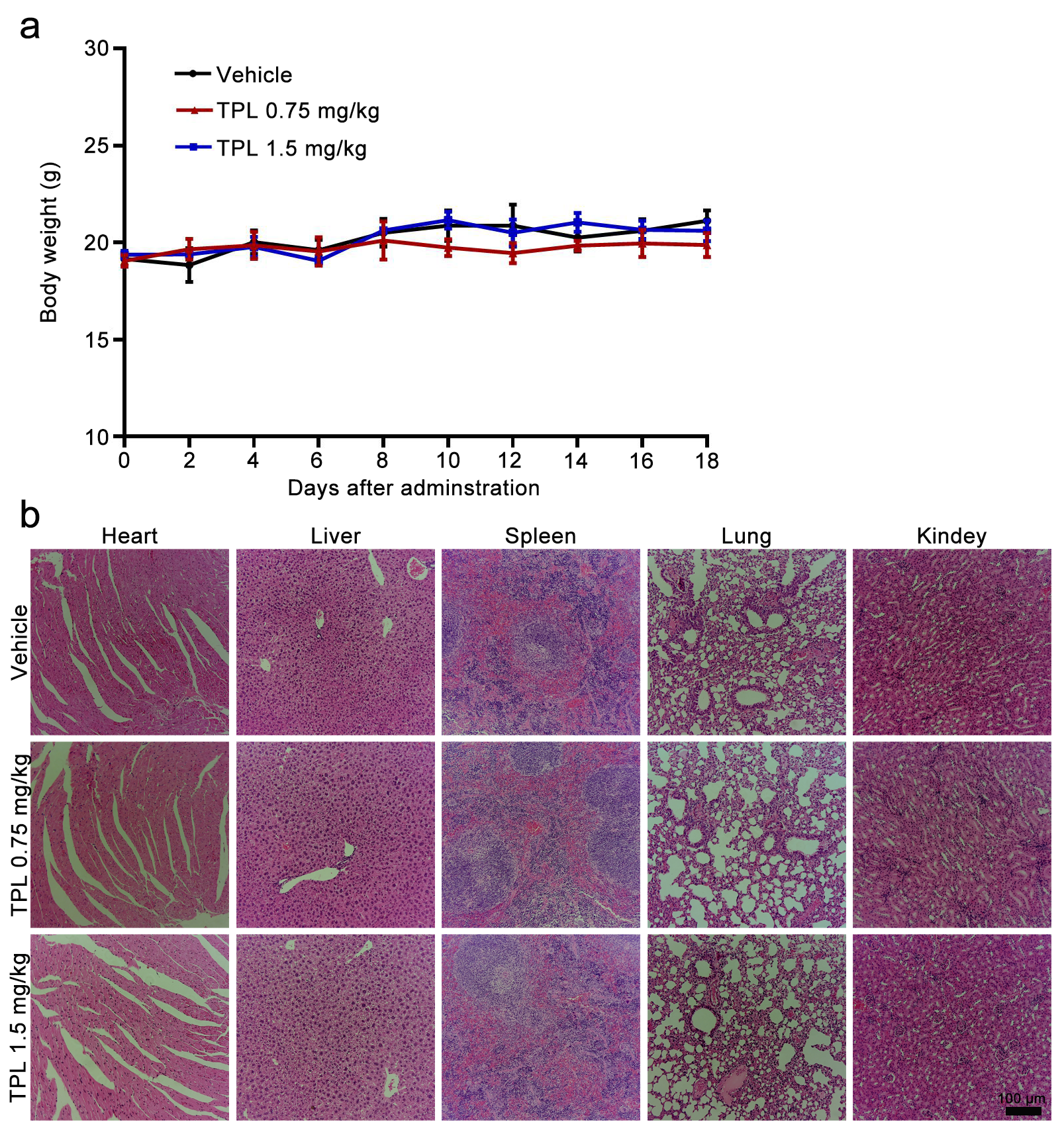

Supplement: Supplementary file 2 — Supplementary Figure 1 [file 41401_2021_657_MOESM2_ESM.tif]

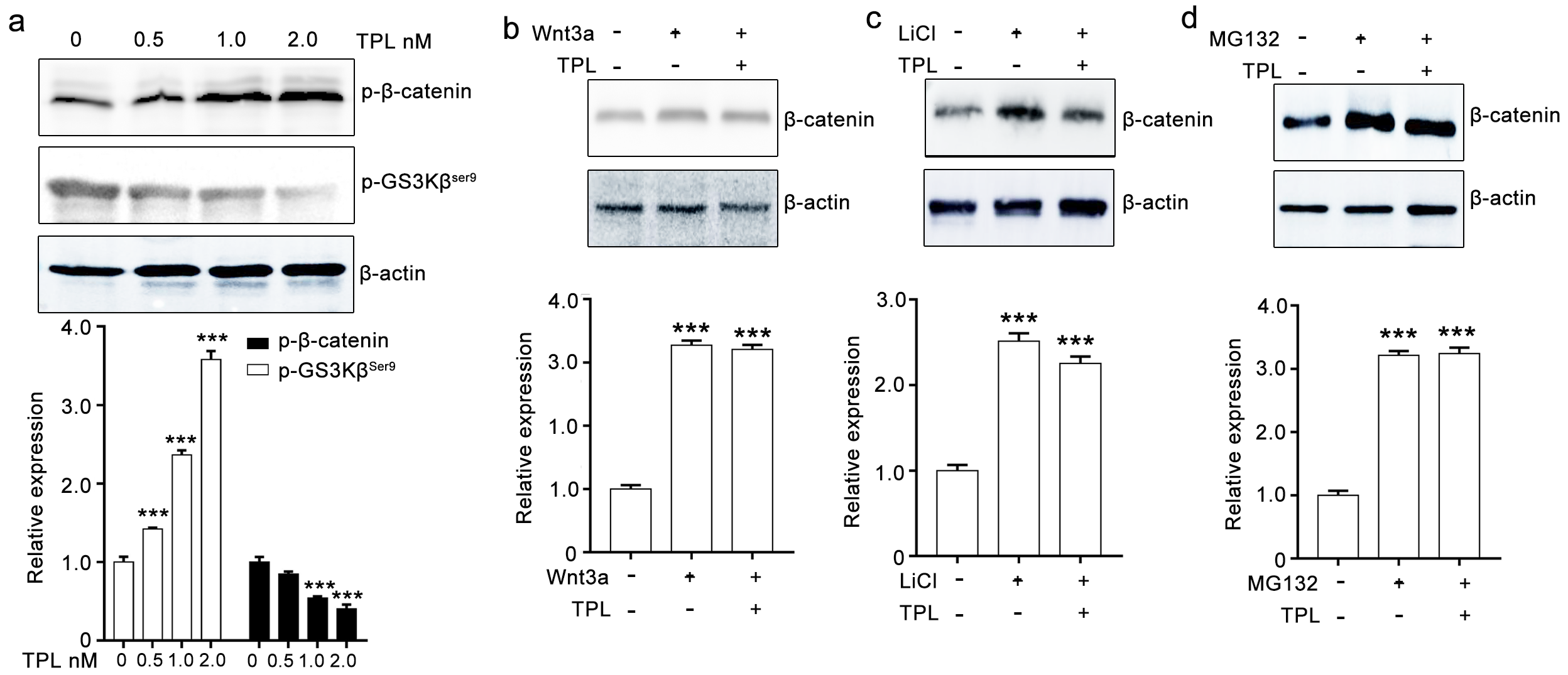

Supplement: Supplementary file 3 — Supplementary Figure 2 [file 41401_2021_657_MOESM3_ESM.tif]
